# T-cell Therapy-Mediated Myocarditis Secondary to Cytokine Release Syndrome

**DOI:** 10.7759/cureus.10022

**Published:** 2020-08-25

**Authors:** Anoshia Afzal, Umar Farooque, Elizabeth Gillies, Lewis Hassell

**Affiliations:** 1 Pathology, University of Oklahoma Health Sciences Center, Oklahoma City, USA; 2 Neurology, Dow University of Health Sciences, Karachi, PAK

**Keywords:** myocarditis, chimeric antigen receptor t-cell therapy, cytokine release syndrome, humans, immune therapy-mediated myocarditis

## Abstract

Chimeric antigen receptor (CAR) T-cell therapy is expanding to a wider patient population; however, cytokine release syndrome (CRS) is the most important adverse event of these therapies. Patients suffering from high-grade CRS also develop signs of cardiac dysfunction and frequently manifest vascular leakage with peripheral and pulmonary edema. We present an unusual case of a 68-year-old female with stage III endometrial carcinosarcoma, who was admitted for T-cell therapy. The patient developed symptoms of CRS within 12 hours of T-cell therapy and expired shortly thereafter. Autopsy of the patient revealed interstitial edema and lymphocytic infiltrates in right and left ventricles along with foci of myocyte necrosis and perivascular fibrosis, more prominent in the right ventricle, consistent with immune therapy-mediated myocarditis. It is important to recognize that CRS progresses rapidly and can have potentially dangerous consequences, so it is imperative to anticipate and treat it early. Cases should be individualized and treated accordingly.

## Introduction

Chimeric antigen receptor (CAR) T-cell therapy is a novel form of immunotherapy approved for the treatment of leukemias and lymphomas by the US Food and Drug Administration (FDA). The most commonly observed side effect of CAR T-cell therapy is cytokine release syndrome (CRS), defined as an inflammatory response leading to the release of massive quantities of cytokines in the body in response to the chimeric T-cell infusion. The clinical manifestations include nausea, vomiting, fever, and chills as well as severe hypotension with tachycardia. T-cell therapies have shown remarkable early success in highly refractory and relapsing malignancies [1]. However, this potent therapy can be accompanied by significant toxicity. CRS and neurotoxicity are the most widely reported, but the true extent and characteristics of cardiovascular toxicity remain poorly understood. The cardiovascular toxicity of CAR T-cell therapy includes adverse clinical outcomes like cardiogenic shock, cardiac arrest, and even cardiac death in rare cases. Here, we have described an unusual case of rapidly progressive myocardial damage and necrosis post CAR T-cell therapy along with a discussion of existing literature and potential side effects of CAR T-cell therapy. We have also discussed possible treatment options to prevent CRS.

## Case presentation

Our patient was a 68-year-old female with progressive stage III endometrial carcinosarcoma who was admitted for T-cell therapy. The patient had a previous history of asthma, alpha galactose intolerance, bilateral mastectomy in 2005 with breast reconstruction in 2006 due to left breast cancer, and total laparoscopic hysterectomy with bilateral salpingo-oophorectomy in 2019. She had no known history of cardiac disease. The patient was admitted for T-cell therapy in May 2020 at our tertiary care hospital. She started having clinical manifestations of CRS within 12 hours of initial dosing. She complained of chest pain and became hypotensive, tachycardia, and hypoxic. A code blue was called and resuscitative efforts began. The patient failed resuscitation and died. A restricted autopsy was performed to rule out cardiac toxicity secondary to T-cell therapy. The heart revealed hypertrophic changes in myocytes of both ventricles along with lipofuscin pigmentation around some nuclei. The right ventricle revealed interstitial edema, scattered mast cells throughout the myocardium, and patchy lymphocytic infiltrate along with focal myocyte necrosis and perivascular fibrosis. The left ventricle had similar findings but to a lesser extent than the right ventricle. Additionally, sections of the lung revealed scattered small bone marrow emboli in the pulmonary vasculature, probably secondary to prolonged chest compression/resuscitation. The overall findings of the autopsy were consistent with immune therapy-mediated myocarditis.

Microscopic sections

**Figure 1:**
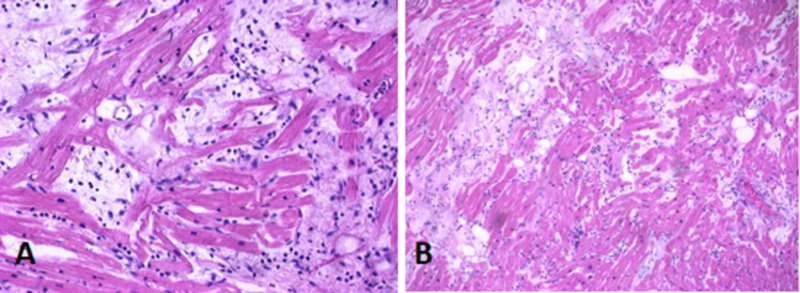
H and E sections of right ventricle (A and B) showing single-cell necrosis with focal interstitial inflammation consisting of macrophages interspersed with lymphocytes, mast cells, and fibroblasts. H and E, hematoxylin and eosin

Myocardial sections showed focal interstitial inflammatory cell infiltration consisting of macrophages, neutrophils, and scattered lymphocytes accompanied by single-cell necrosis. Other areas showed inflammatory cells within capillaries and extracapillary also associated with contraction band necrosis, likely a terminal event secondary to catecholamine-induced necrosis. The changes within the heart sections were consistent with single-cell necrosis with inflammation, which was mostly composed of macrophages, neutrophils, and some lymphocytes (Figure 1).

**Figure 2:**
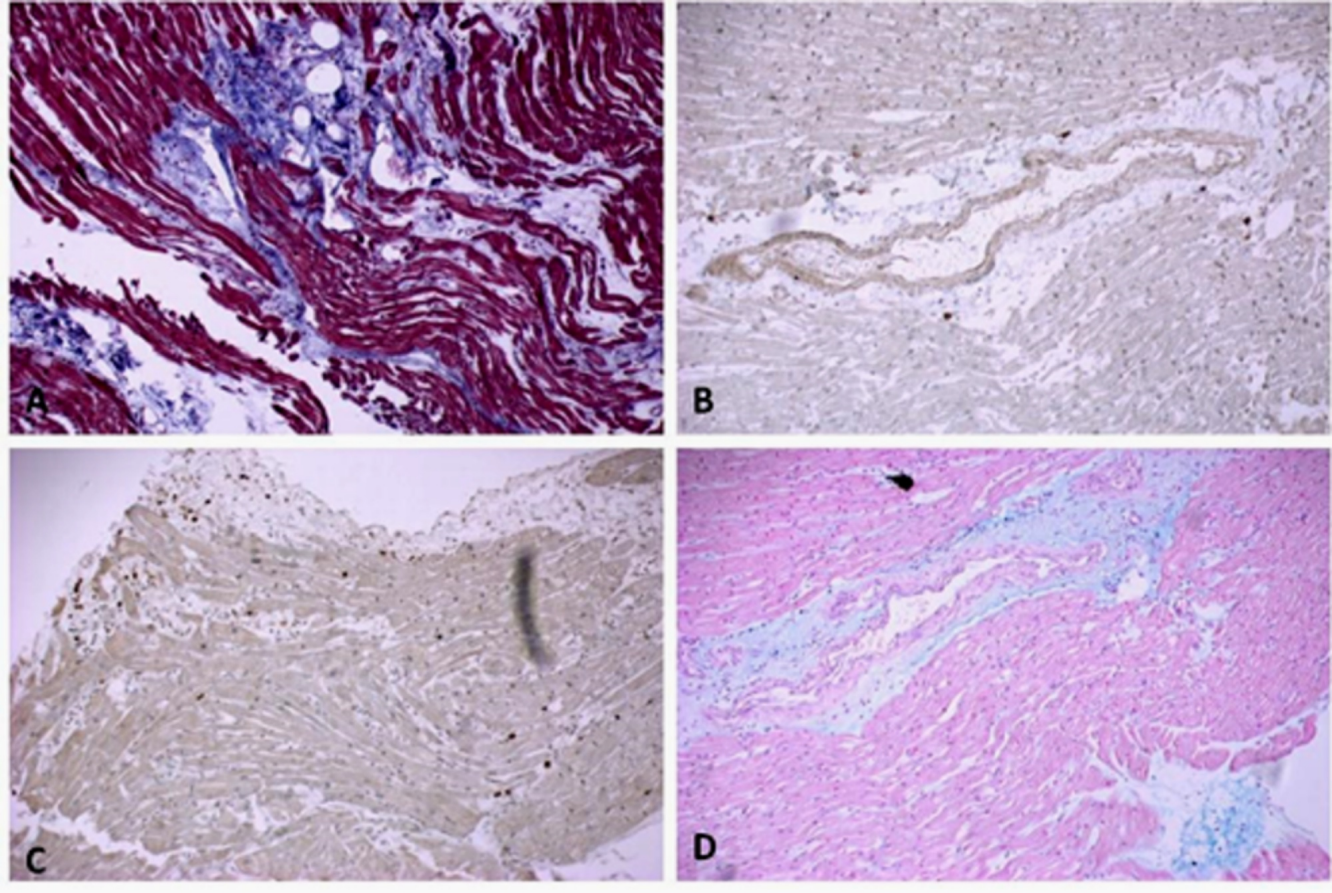
(A) Trichrome highlights perivascular and interstitial fibrosis and areas of myocyte necrosis in right ventricle. Note the purple cells highlighting single cell necrosis in an area of inflammation. (B) CD117-positive cells were frequently observed that highlight scattered mononuclear/mast cells. (C) CD3-positive T-cells are seen scattered throughout the myocardium; however, no clusters of T cells are present. (D) Alcian blue highlights scattered mononuclear/mast cells CD, cluster of differentiation

By immunostaining, cluster of differentiation (CD) 3 cells were observed scattered within the myocardium in sections from the right ventricle but no clusters of T cells were observed (Figure 2).

The changes observed in the heart were consistent with a vasopressor (catecholamine)-induced single-cell necrosis, which was most prominent in the right ventricle and less in the left ventricle sections.

**Figure 3:**
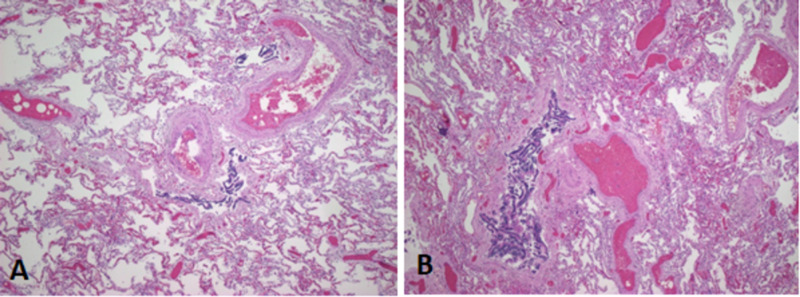
Sections of the right middle and lower lobes of lung (A and B) showing platelet thrombi in muscular arteries along with bone marrow embolus.

Lung sections showed multiple foci of platelet thrombi without any organization and early changes of intimal thickening in some muscular arteries. Right upper lobe sections showed hemorrhage and necrosis with scattered acute inflammatory cells (small infarct) (Figure 3).

## Discussion

CAR T-cell therapy causes cytokine-associated toxicity in the form of CRS. Immune cells involved in the pathogenesis include B and T lymphocytes, natural killer cells, and myeloid cells, such as macrophages, dendritic cells, and monocytes [1,2]. A large number of cytokines are released that mediate a hyperimmune response. Some of these cytokines include interleukin (IL)-2, soluble IL-2R, interferon-gamma, IL-6, soluble IL-6R, and granulocyte-macrophage colony-stimulating factor; IL-6 being a very significant cytokine for CRS. This can result in several symptoms based on the severity and grade of CRS. It can also cause organ toxicities, involving the cardiovascular, nervous, respiratory, gastrointestinal, hepatic, renal, and hematological systems [3].

The cardiac complications of CRS are poorly understood but are related to severe CRS causing myocardial toxicity and vascular leaks with hypotension. Therefore, cardiovascular toxicity findings include hypotension, tachycardia, edema, and hypoproteinemia [2]. T-cell proliferation causes elevated levels of IL-6 even in the myocardium and pericardial fluid.

CAR T-cell therapy is widely used as a treatment strategy for B-cell hematological malignancies, such as B-cell precursor acute lymphoblastic leukemia and B-cell lymphoma [4]. It has been particularly used for refractory and relapsing cases [2]. The CAR is a recombinant fusion protein that recognizes tumor antigens and is capable of activating T cells in a major histocompatibility complex (MHC) unrestricted manner. Autologous T cells are first collected from the patient, followed by viral-mediated integration of CAR into the host genome. For this purpose, the commonly used viral vectors are retroviruses such as the human immunodeficiency virus (HIV)-based lentiviral vectors [5]. T-cell therapy has structurally gone through three generations with the integration of intracellular domains with costimulatory signaling domains [6].

The management of CRS depends on the grading as published by the American Society for Transplantation and Cellular Therapy [7]. Low-grade CRS is usually treated with supportive therapy such as antipyretics and antiemetics because the symptoms are not life threatening and mostly include fever, nausea, fatigue, and myalgia. More severe cases are treated with IL-6 blockers, keeping in mind the important role of cytokines such as IL-6 in the severity of CRS. Tocilizumab is an IL-6 receptor targeting monoclonal antibody and siltuximab is a chimeric anti-IL-6 monoclonal antibody that blocks IL-6 signaling by binding to soluble IL-6. The use of tocilizumab, approved by the FDA, has become a standard of care in treating CRS, while siltuximab has not been as extensively used [8]. Corticosteroid therapy is also administered as second-line therapy in case the IL-6 blockade is ineffective. The use of tocilizumab remains the first-line therapy in patients having CRS grade II or higher [3,9,10].

The prevalence of data regarding cardiovascular complications is rare. The first study in 2018 included 98 patients to determine the potential cardiovascular toxicities associated with CD19 CAR T-cell therapy [11]. Twenty-four patients developed hypotension requiring ionotropic support due to a high disease burden and a pre-existing cardiac dysfunction. Twenty-one patients developed life-threatening hypotension requiring tocilizumab alone or with steroids. Persistent systolic dysfunction was rare in patients. Hemodynamic instability with hypotension was seen again in a study with patients having relapsed or refractory chronic lymphocytic leukemia [12]. Another study by Fitzgerald et al. enrolled 39 subjects with relapsed/refractory acute lymphoblastic leukemia in which cardiovascular dysfunction was seen in 36% of the patients [13].

## Conclusions

Cardiovascular toxicity observed with the CAR T-cell therapy is rare and is usually associated with CRS. Therefore, all patients should be checked for the cardiotoxicity profile of CAR T-cell therapy to design essential primary and secondary prevention strategies and to minimize the occurrence and progression of cardiovascular complications and toxicity.

## References

[1] Lee DW, Gardner R, Porter DL (2014). Current concepts in the diagnosis and management of cytokine release syndrome. Blood.

[2] Ghosh AK, Chen DH, Guha A, Mackenzie S, Walker JM, Roddie C (2020). CAR T cell therapy-related cardiovascular outcomes and management: systemic disease or direct cardiotoxicity?. J Am Coll Cardiol.

[3] Neelapu SS, Tummala S, Kebriaei P (2018). Chimeric antigen receptor T-cell therapy—assessment and management of toxicities. Nat Rev Clin Oncol.

[4] Brown CE, Mackall CL (2019). CAR T cell therapy: inroads to response and resistance. Nat Rev Immunol.

[5] Salter AI, Pont MJ, Riddell SR (2018). Chimeric antigen receptor-modified T cells: CD19 and the road beyond. Blood.

[6] Maude S, Barrett DM (2016). Current status of chimeric antigen receptor therapy for haematological malignancies. Br J Haematol.

[7] Lee DW, Santomasso BD, Locke FL (2019). ASTCT consensus grading for cytokine release syndrome and neurologic toxicity associated with immune effector cells. Biol Blood Marrow Transplant.

[8] Subklewe M, von Bergwelt-Baildon M, Humpe A (2019). Chimeric antigen receptor T cells: a race to revolutionize cancer therapy. Transfus Med Hemother.

[9] June CH, Sadelain M (2018). Chimeric antigen receptor therapy. N Engl J Med.

[10] Santomasso B, Bachier C, Westin J, Rezvani K, Shpall EJ (2019). The other side of CAR T-cell therapy: cytokine release syndrome, neurologic toxicity, and financial burden. Am Soc Clin Oncol Educ Book.

[11] Burstein DS, Maude S, Grupp S, Griffis H, Rossano J, Lin K (2018). Cardiac profile of chimeric antigen receptor T cell therapy in children: a single-institution experience. Biol Blood Marrow Transplant.

[12] Porter DL, Hwang WT, Frey NV (2015). Chimeric antigen receptor T cells persist and induce sustained remissions in relapsed refractory chronic lymphocytic leukemia. Sci Transl Med.

[13] Fitzgerald JC, Weiss SL, Maude SL (2017). Cytokine release syndrome after chimeric antigen receptor T cell therapy for acute lymphoblastic leukemia. Crit Care Med.

